# Online Career Master of Public Health Program for Working Professionals

**Published:** 2013-03-08

Designed for working professionals who are unable to leave their jobs, the Career Master of Public Health (CMPH) program at the Rollins School of Public Health, Emory University, is a distance-learning program. Each semester, students are required to attend two long weekends on the Emory University campus in Atlanta, Georgia. All additional coursework is taught using the Internet and distance-learning techniques.

The CMPH program offers three majors: Applied Epidemiology, Applied Public Health Informatics, and Prevention Science. In addition to coursework in their major, students in the CMPH program take six required core courses (epidemiology, biostatistics, social behavior, environmental health, global health, and the U.S. health-care system) and additional coursework that includes competencies in public health informatics, evaluation, and surveillance. All CMPH students also complete a practicum and culminating experience that are relevant to their areas of specialization.

Program faculty members represent both academia and public health practice and include persons who work at Emory, CDC, and local public health departments. Completion of coursework for the CMPH degree takes 7 semesters for the full-time student and is fully accredited by the Council on Education for Public Health and the Southern Association of Colleges and Schools. Prospective students should apply by May 1, 2013, to guarantee consideration for the fall 2013 semester. Additonal information is available via e-mail at cmph@sph.emory.edu or visit online at http://www.sph.emory.edu/cmph.

